# A BSL-2 chimeric system designed to screen SARS-CoV-2 E protein ion channel inhibitors

**DOI:** 10.1128/jvi.02252-24

**Published:** 2025-04-30

**Authors:** Vashi Negi, Richard J. Kuhn

**Affiliations:** 1 Department of Biological Sicences, Purdue University 311308 , West Lafayette, Indiana, USA; 2 Purdue Institute of Inflammation, Immunology, and Infectious Disease, Purdue University 311308 , West Lafayette, Indiana, USA; University of North Carolina at Chapel Hill, Chapel Hill, North Carolina, USA

**Keywords:** viroporins, sindbis virus, SARS-CoV-2, envelope protein, ion channel inhibitors, alphavirus 6K, chimeric viruses

## Abstract

A major hindrance to the identification of new drug targets and the large-scale testing of new or existing compound libraries against severe acute respiratory syndrome coronavirus 2 (SARS-CoV-2) is that research on the virus is restricted to biosafety level 3 (BSL-3) laboratories. In such cases, BSL-2 surrogate systems or chimeric and attenuated versions of the virus are developed for safer, faster, and cheaper examination of the stages of the virus life cycle and specific drug targets. In this study, we describe a BSL-2 chimeric viral system utilizing a Sindbis virus background as a tool to study one such target, the SARS-CoV-2 Envelope (E) protein channel activity. This protein is fully conserved between SARS-CoV and SARS-CoV-2 variants of concern (VOCs), except for a threonine to isoleucine mutation in the Omicron variant, making the E ion channel domain an attractive antiviral target for combination therapy. Using a BSL-2-chimeric system, we have been able to show similar inhibition profiles using channel inhibitors as previously reported for E-channel inhibition in authentic SARS-CoV-2. This system has the potential to allow faster initial screening of E-channel inhibitors and can be useful in developing broad-spectrum antivirals against viral channel proteins.

**IMPORTANCE:**

Despite its importance in viral infections, no antivirals exist against the ion channel activity of the SARS-CoV-2 Envelope (E) protein. The E protein is highly conserved among SARS-CoV-2 variants, making it an attractive target for antiviral therapies. Research on SARS-CoV-2 is restricted to BSL-3 laboratories, creating a bottleneck for screening potential antiviral compounds. This study presents a BSL-2 chimeric system using a Sindbis virus background to study the ion channel activity of the E protein. This novel BSL-2 system bypasses this limitation, offering a safer and faster approach for the initial screening of ion channel inhibitors. By replicating the channel inhibition profiles of authentic SARS-CoV-2 in a more accessible system, this research paves the way for the development of broad-spectrum antivirals against viral channel proteins, potentially expediting the discovery of life-saving treatments for COVID-19 and other viral diseases.

## INTRODUCTION

The emergence of the novel severe acute respiratory syndrome coronavirus 2 (SARS-CoV-2), in December 2019, started a global pandemic infecting more people than the preceding outbreaks of related coronaviruses, SARS-CoV and Middle East respiratory syndrome coronavirus (MERS-CoV) (1, 2). COVID-19, the disease caused by SARS-CoV-2 infection, has resulted in over 700 million cases of infection and over 7 million deaths reported worldwide as of September 2024 (3, 4). Apart from developing and administering vaccines in record time, a few treatment options have been made available to patients under the Emergency Use Authorization (EUA) and European Medicines Agency (EMA) advice (5, 6) such as monoclonal antibodies, remdesivir, Paxlovid, and molnupiravir (7–13). Other repurposed drugs and intervention strategies are being tested in over 9,000 ongoing clinical trials (14). While the COVID-19 public health emergency expired in May 2023, COVID-19 continues to be a major global health concern. The highly transmissible Omicron variant and its subvariants have acquired mutations in the Spike protein receptor-binding domain (RBD) that allow immune evasion from both monoclonal antibodies and vaccines (15, 16). While effective vaccines remain our best option against fighting this disease long term, there is a need to find better antiviral drugs and drug targets to generate treatment options that can be effective against emerging variants to help prevent severe disease outcomes in infected patients. To aid in the initial screening of compound libraries against a promising target, the ion channel activity of the SARS-CoV-2 E protein, we have developed a BSL-2 chimeric system that uses Sindbis virus (SINV) as the background.

Viroporins are small, oligomeric hydrophobic viral proteins that permeabilize host cell membranes by forming pores, thereby disrupting normal cellular functions and assisting in viral infection (17). These proteins have certain common structural and functional features and can conduct ions (mainly cations) and small molecules through them (18). Viroporins such as influenza A virus M2 (IAV-M2) and alphavirus 6K (6 kilodalton) have been studied extensively as potential drug targets owing to their importance in viral entry and exit (19, 20). IAV-M2 is a class I single-pass membrane protein that regulates viral entry and uncoating (17, 21). It also mediates glycoprotein trafficking and virus release at the plasma membrane through its ion-channel domain (22, 23). Although much less is known about the function of alphavirus 6K compared to IAV-M2, studies done in *Xenopus* oocytes and planar lipid bilayers have shown that 6K has ion-channel activity and is highly toxic when produced in bacterial cells (24–26). Unlike M2, the structure and oligomeric state of 6K are unknown. Using a glycosylation-based translocation assay, SINV 6K was recently shown to span the host membrane once, although past studies had predicted two transmembrane domains (27, 28). The transmembrane helix of 6K is associated with ion channel activity while the C-terminal sequence acts as a translocation signal for glycoprotein E1 that follows 6K in the polyprotein sequence (25, 29). Interestingly, 6K has a conserved ribosomal frameshift site downstream of the transmembrane domain that leads to the synthesis of the TransFrame (TF) protein that has the same N-terminal sequence as 6K but a different C-terminus sequence (30). The exact function of TF or the implications of this frameshift event on the viral life cycle is not fully understood yet, but both 6K and TF share the ion-channel transmembrane domain. For simplicity, 6K and TF will both be addressed as “6K” with respect to their ion-channel property in the remainder of this report. It has long been known that 6K is involved in viral budding, and recently this function, along with the trafficking of glycoproteins and the biogenesis of cytopathic vacuoles CPV-II have been attributed to the ion-channel activity of 6 K (31, 32). Notwithstanding, 6K is not indispensable for the growth and production of alphaviruses (30, 32).

E protein is the smallest structural protein of SARS-CoV-2 and is classified as a viroporin due to its ability to form pores and ion channels in host cell membranes (33). Previous studies on SARS-CoV E protein attributed its ion channel activity to the transmembrane region of the protein (34, 35). E protein is highly conserved among ꞵ-coronaviruses with the transmembrane (TM) domain being completely conserved between SARS-CoV E and SARS-CoV 2 E proteins (36). In 2020, the structure of the TM domain of the SARS-CoV-2 E protein was solved as a pentameric cation-selective channel sensitive to hexamethylene amiloride (HMA) (37). Similar to other viroporins, SARS-CoV E protein is permeable to cations and can disrupt Ca^2+^ homeostasis leading to the activation of host inflammasomes (38). Recently, the expression of SARS-CoV-2 E protein was shown to induce robust immune responses and cell death *in vitro* and *in vivo* (39). The effect seen was similar to the cytokine storm and acute respiratory distress syndrome (ARDS)-like damage reported in COVID-19 patients. This points toward a role for the protein as a virulence factor during infection, making it a promising drug target for antivirals. Indeed, studies have shown that blocking the E channel by channel inhibitors leads to a decrease in viral infection levels of SARS-CoV-2 (39, 40).

To develop a BSL-2 virus system for studying inhibition of E-channel activity, we designed a chimeric SINV, replacing the ion channel transmembrane helix of 6K protein with that of E protein (ETM), as previously described in Elmasri et al. (32) for other viroporins, except the frame-shift site in the 6K sequence was not disturbed. Hence, the chimeric virus would produce both 6K and TF proteins with ETM replacing their ion channel TM domain. This chimera was tested for functional complementation to assess whether the resultant rescue of viral titer was a result of a functional ETM ion channel in SINV. We used known channel inhibitors such as amantadine and amiloride derivatives to test inhibition in the chimeric system and compare it to inhibition in the authentic SARS-CoV-2. Amantadine is a well-known channel inhibitor that was first used as an FDA-approved antiviral against influenza (41, 42). The exact mechanism of action was later understood to be one of interfering with the ion channel activity of the M2 proton channel in the virus particle (19). However, due to drug resistance, the drug failed to remain effective in treating Influenza and was discontinued as a treatment option (43). Amantadine is also known to inhibit the channel activity of SARS-CoV ETM in lipid bilayers (34) and of SARS-CoV-2 E in *X. laevis* oocyte expression system (44). Derivatives of the FDA-approved antikaliuretic drug amiloride (“midamor”) such as HMA, EIPA (ethyl isopropyl amiloride), and DMA (dimethyl amiloride) are also known inhibitors of ion channel activity that show antiviral effect during SARS-CoV-2 infection (40).

Using an mCherry SINV reporter virus previously described by Jose et al. (45) for quantification of viral infection levels, we have demonstrated comparable inhibition profiles and trends of these compounds in this chimeric system and the authentic virus. Our system has the potential to assist in faster screening for the discovery of new E inhibitors. It can also be used to provide more insight into the role of the protein in the viral life cycle and serve as a tool for developing COVID-19 intervention strategies in the future.

## MATERIALS AND METHODS

### Viruses and cells

BHK-21 (baby hamster kidney) cells were maintained at 37°C and 5% CO_2_ in Eagle’s minimum essential medium (MEM) (Gibco) supplemented with 10% heat-inactivated fetal bovine serum (FBS, Sigma-Aldrich). Vero cells were maintained in Dulbecco’s modified Eagle medium (DMEM) (Gibco) supplemented with 10% FBS. A full-length cDNA clone pToto64 (46) was used for generating wild-type and mutant SINV.

### Plasmids and cloning

The codon-optimized sequence of the full-length SARS-CoV-2 E protein (NCBI reference sequence MN985325.1) was synthesized by GENEWIZ, Inc. (South Plainfield, NJ). Full-length 6K protein sequence was isolated from pToto64 using PCR. The sequences of 6K and E were cloned into cloning vector pET His6 MBP N10 TEV (Addgene #29706) using In-fusion cloning and transformed into DH5alpha cells for making glycerol stocks. Another plasmid was generated expressing EGFP (enhanced green fluorescent protein) in the place of the MBP-TEV sequence in #29706 with In-fusion cloning. Δ6K SINV was generated by making a deletion mutation in pToto64 as described in Elmasri et al. (32). SINV-ETM and its channel-inactivating mutant chimeras (with the single mutations N15A and V25F and the double mutation N15A, V25F) were generated using the Q5 Site-directed mutagenesis kit (New England Biolabs #E0554S) to introduce 8–38 residues of E protein TM domain (ETGTLIVNSVLLFLAFVVFLLVTLAILTALR) (37) in pToto64. These mutations were also made in the mCherry-tagged E2 SINV background described in Jose et al. (45) where the fluorescent tag is added to the N-terminus of E2 after the E3-E2 furin cleavage site. All of the altered sequences were confirmed by sequencing.

### *In vitro* transcription and transfection

Plasmids were linearized with SacI and transcribed *in vitro* using SP6 RNA polymerase as previously described in Tang et al. (47) to produce viral RNA. BHK-21 cells were transfected with 10 µg of RNA using electroporation and used to produce and isolate single plaques. Virus from single plaques was grown to produce viral stocks that were titered in BHK-21 cells.

### Plaque assays

Virus stocks were serially diluted in MEM and added to BHK-21 cell monolayers grown on six-well plates. Cells were overlaid with 1% agarose, MEM, and 5% FBS after 1 hour of rocking at room temperature to allow the virus to adsorb to cells. Plates were stained with neutral red (MilliporeSigma, #N2889) diluted in phosphate-buffered saline (PBS) after 48 hours of incubation at 37°C. The virus titers were determined by manually counting the number of plaques. All assays were done in triplicate.

### Growth curve analyses

BHK-21 cells grown in six-well plates were infected with the stock virus at a multiplicity of infection (MOI) of 1.0 or 2.0 and 0.1 for one-step and continuous growth kinetic studies, respectively. Plates were rocked for 1 hour at room temperature followed by incubation at 37°C. For one-step growth curve analysis, spent media from each well containing cells was removed, and the wells were washed extensively with PBS before adding fresh media. This was done 1 hour prior to each harvest. Infectious virus titer was determined by plaque assays performed on a monolayer of BHK-21 cells. Two independent experiments for one-step growth curve analyses of each virus were conducted with three technical replicates in each experiment. For continuous growth curve analysis, 100 µL of supernatant was harvested at given time points and replaced with 100 µL of fresh media. The harvested supernatants were used to perform plaque assays. The experiment was carried out once with three technical replicates.

### qRT-PCR and specific infectivity

A quantitative real-time PCR was performed as previously described (32). BHK-21 cells were electroporated with *in vitro* transcribed viral RNA and incubated at 37°C. Media were removed and cells were washed thrice with PBS 4 hours after electroporation. Fresh media was added, and the cells were placed in the incubator for an additional 8 hours. 12 hours post-electroporation, RNA from the supernatant was harvested and purified using the Qiagen RNeasy Mini kit (#74104). qRT-PCR was performed in triplicate for each sample using SuperScript III Platinum SYBR Green One-Step qRT-PCR kit with ROX (ThermoFisher #11746100) and SINV nsp1-specific primers 5′ TTCCCTGTGTGCACGTACAT 3′ and 5′ TGAGCCCAACCAGAAGTTTT 3′. *In vitro* transcribed RNA for wild-type SINV of known concentrations was used to generate a standard curve which was then used to determine the number of RNA copies in each sample. The viral supernatants were further used to measure titers using plaque assays and to calculate specific infectivity for each virus as the ratio of the number of RNA genomes to the virus titer in each sample.

### Protein expression and purification

Bacterial clones of full-length SINV 6K, SARS-CoV-2 E, EGFP, and E mutant proteins (N15A, V25F, and N15A, V25F double mutant) in pET His6 MBP N10 TEV (Addgene #29706) were used for bacterial expression and purification. The MBP-TEV region was deleted for EGFP, E, and E mutant constructs so that only SINV 6K has a N-terminal MBP-tag. These plasmids were confirmed by sequencing and individually transformed into BL21-CodonPlus (DE3)-RIL cells for overexpression. A single colony was picked for overnight small-scale culture and then inoculated in 750 mL TB medium. The cells were induced by adding 0.5 mM isopropyl b-D-thiogalactopyranoside (IPTG) and grown at 16°C overnight.

The cells expressing 6K, E, and E mutants were harvested and then resuspended in lysis buffer (25 mM Tris-HCl pH 7.6, 100 mM NaCl, 5 mM imidazole, 1% Triton X-100, 1 mM CaCl_2_, 1 mM MgCl_2_, 1 mM phenylmethylsulfonyl fluoride [PMSF], 5 mM 2-mercaptoethanol [BME], 10 µg/mL DNase I and PierceTM EDTA-free protease inhibitor [1 tablet per 100 mL lysate]). After sonication and centrifugation, the supernatants were incubated with Ni-NTA resin for 1 hour at 4°C with gentle stirring. The resin was washed with wash buffers I and II containing 10 mM and 30 mM imidazole, respectively, along with 0.1% n-dodecyl-β-D-maltopyranoside (DDM) (Anatrace D310S). The protein was eluted with elution buffer (25 mM Tris-HCl, pH 7.6, 100 mM NaCl, 1 mM TCEP, 300 mM imidazole, and 0.015% DDM). TEV protease was added to His-MBP-6K fusion protein eluate at a 1:10 (protease:protein) ratio to remove the His-MBP tag and placed in a 3 kDa cut dialysis bag overnight to remove excess imidazole. The dialyzed protein samples were cleaned up using a Ni-NTA affinity column, and flowthrough was collected. His-E and His-E mutant protein eluates were also dialyzed for imidazole removal. The target proteins were then concentrated and further purified by size exclusion (Superdex 200, GE Healthcare) chromatography (SEC) for final elution in SEC buffer (25 mM Tris-HCl pH 7.6, 100 mM NaCl, 0.015% DDM, 5 mM DTT, 1 mM CaCl_2_, 1 mM MgCl_2_). Samples corresponding to different peak fractions were pooled and concentrated using a 3 kDa MWCO concentrator (Millipore Sigma) to 0.5–1 mg/mL. These samples were diluted in Laemmli loading buffer, boiled for 10 minutes at 95°C, and run on 4%–20% SDS-PAGE gels (Bio-Rad).

Bacterial cells expressing EGFP were harvested and then resuspended in lysis buffer (25 mM Tris-HCl pH 7.6, 1 M NaCl, 5 mM imidazole, 1 mM PMSF, 5 mM BME, and 10 µg/mL DNase I). The cells were then sonicated and centrifuged for 1 hour incubation of the supernatant with Ni-NTA resin at 4°C. Wash buffers I and II containing 10 mM and 30 mM imidazole without any detergent were used to wash the resin. The protein was eluted with elution buffer (25 mM Tris-HCl, pH 7.6, 500 mM NaCl, 5 mM BME, and 300 mM imidazole). The EGFP eluate was dialyzed to remove imidazole and concentrated for SEC (25 mM Tris-HCl pH 7.6, 150 mM NaCl, 5 mM DTT). Fractions corresponding to the EGFP peak were concentrated and used for SDS-PAGE analysis and inhibitor binding experiments.

### Western blot analysis

A transfer blot at 90V for 100 minutes was used to transfer the bands on an SDS-PAGE gel onto a nitrocellulose membrane. Blocking was done using 3% bovine serum albumin (BSA) (Fisher Scientific #BP9700100) followed by the addition of the primary antibody—rabbit anti-E antibody (Abcam #AB272503) against SARS-CoV-2 E. Goat anti-rabbit IgG (Abcam #AB205718) was used as the secondary antibody. The signal was detected using chemiluminescence (ThermoFisher #32109).

### Inhibitor binding analysis

HMA, EIPA, and DMA ion channel inhibitor compounds (Millipore Sigma A9561, A3085, A4562) were dissolved in 100% methanol to make 4 mM stock solutions. Purified SINV 6K and SARS-CoV-2 E and mutant proteins (1.5 mg/mL in SEC buffer) were incubated with 3–4 × molar excess amounts of the compounds for 1 hour on ice. Samples were dialyzed in a 3 kDa cut dialysis bag overnight at 4°C to remove unbound compounds. Equivalent amounts of protein in methanol and compounds in SEC buffer were used as controls. Samples were then loaded onto a 96-well plate in equal amounts and screened for absorbance profiles using an iD5 spectrophotometer (Molecular Devices). Two independent experiments were performed, and absorbance values were plotted for each wavelength using GraphPad Prism software to generate absorbance spectra for inhibitor-bound proteins.

### Cytotoxicity assay

100 mM stocks of HMA, EIPA, DMA, and 1 M stock of Amantadine were made in DMSO (Millipore Sigma D2650). BHK-21 and Vero cells were seeded into 96-well plates (Falcon Corning) and treated with the indicated concentrations of the compounds for the specified time periods at 37°C. Cytotoxicity assays were conducted using a Calorimetric Cell Proliferation kit-based assay with WST-1 (water-soluble Tetrazolium salt) as the substrate (BioVision). After 3–4 hour incubation with WST-1, absorbances at 440 nm and 650 nm (reference) were measured on an iD5 Spectrophotometer. Two independent experiments were performed in duplicate. All the readings were plotted using a standard line curve to interpolate CC_50_ values using GraphPad Prism.

### Efficacy assay

BHK-21 cells were seeded in 96-well plates. At t = 0, the cells were treated with different concentrations of the compounds diluted in stock mCherry-tagged viruses at a final MOI of 1. 0.1% DMSO was used as control, and cells were incubated at 37°C for 9 to 12 hours. After 12 hours, the mCherry signal was quantified using fluorescence spectroscopy (excitation wavelength = 590 nm, emission wavelength = 635 nm) on an iD5 plate reader and used as a measurement of viral growth. Percentages of average absorbance values from wells scanned relative to the DMSO controls were used to plot IC_50_ graphs using GraphPad Prism software. A non-linear regression curve model for inhibitor vs. response (variable slope, 4-parameter) was used to analyze the graphs and determine IC_50_ values. The assay was performed twice for WT SINV, ∆6K SINV, and SINV-ETM, and once for the E-channel mutants (N15A, V25F, N15A, and V25F double mutant) in duplicate for each concentration of the inhibitors. To validate iD5 readings, supernatants of untagged viruses were collected after 12 hours to determine the reduction in viral titer due to compound inhibition using plaque assays. Images of mCherry-tagged viruses and DAPI-stained cells were taken after 12 hours of incubation with compounds using a Cytation 7 plate reader. IC_50_ values were also determined for SINV and SINV-ETM infected at an MOI of 0.1 in the presence of inhibitors 24 hours post-infection using the mCherry signal.

### Egress inhibition assay

BHK-21 cells were infected with wild-type SINV, SINV-ETM, and ∆6K SINV at an MOI of 5 in a 96-well plate. After 8 hours of incubation at 37°C, the virus inoculum was removed, and the cells were washed thrice with PBS. Indicated concentrations of the compounds and DMSO control were added to the cells, and infection was allowed to continue for an additional 4 hours. After 12 hours of infection, the cell supernatants were harvested and plaqued on BHK-21 cells. Viral titers were determined from two independent experiments and plotted using GraphPad Prism.

### Time of inhibitor addition assay

BHK-21 cells were seeded in a 96-well plate and treated with indicated concentrations of channel inhibitors (>IC_50_) at different time points before, during, and after the addition of the virus. At t = 0, mCherry-tagged viruses (wild-type SINV and SINV-ETM) were diluted in low serum media (MEM containing 2% FBS) and added to cells at MOIs of 0.1 (low MOI) and 10 (high MOI). 25 µM of HMA and EIPA, 50 µM of DMA, and 500 µM of Amantadine were added to the cells at t = −1, 0, 1, 4, 6, 8, 10, and 12 h time points. Two independent experiments were performed for every condition with 0.1%–0.2% DMSO as the control. Cells were fixed with 4% PFA at t = 15 h post-infection, stained with DAPI, and imaged using a Cytation 7 image reader (Agilent BioTek) in grayscale.

For cells infected at the MOI of 10, the mCherry fluorescence signal was quantified using the Gen5 software for Cytation 7. Percent relative infection was calculated by taking the ratio of mCherry fluorescence signal values for compound-treated vs. DMSO-treated cells at the same time point and multiplying by 100. Data from two independent experiments were then plotted using GraphPad Prism with error bars representing the standard error of the mean (SEM). A two-tailed unpaired t-test was performed to assess statistical significance (*P* < 0.05) between the fluorescence signal values of the treated and control samples at each time point for SINV and SINV-ETM chimera-infected cells.

### Plaque number reduction assay

Known titers of WT SINV and ∆6K SINV were preincubated with different concentrations of HMA (6 nM, 0.39 µM, and 25 µM) and PBS control for 1 hour at 37°C. The samples were then filtered using 100 kDa-cutoff centrifugal filters (Amicon) to remove the unbound compounds, and plaque assays were performed right after filtration. The experiment was performed twice.

## RESULTS

### SARS-CoV-2 E protein TM domain can partially replace the function of the ion channel domain of 6K in SINV

**Fig 1:**
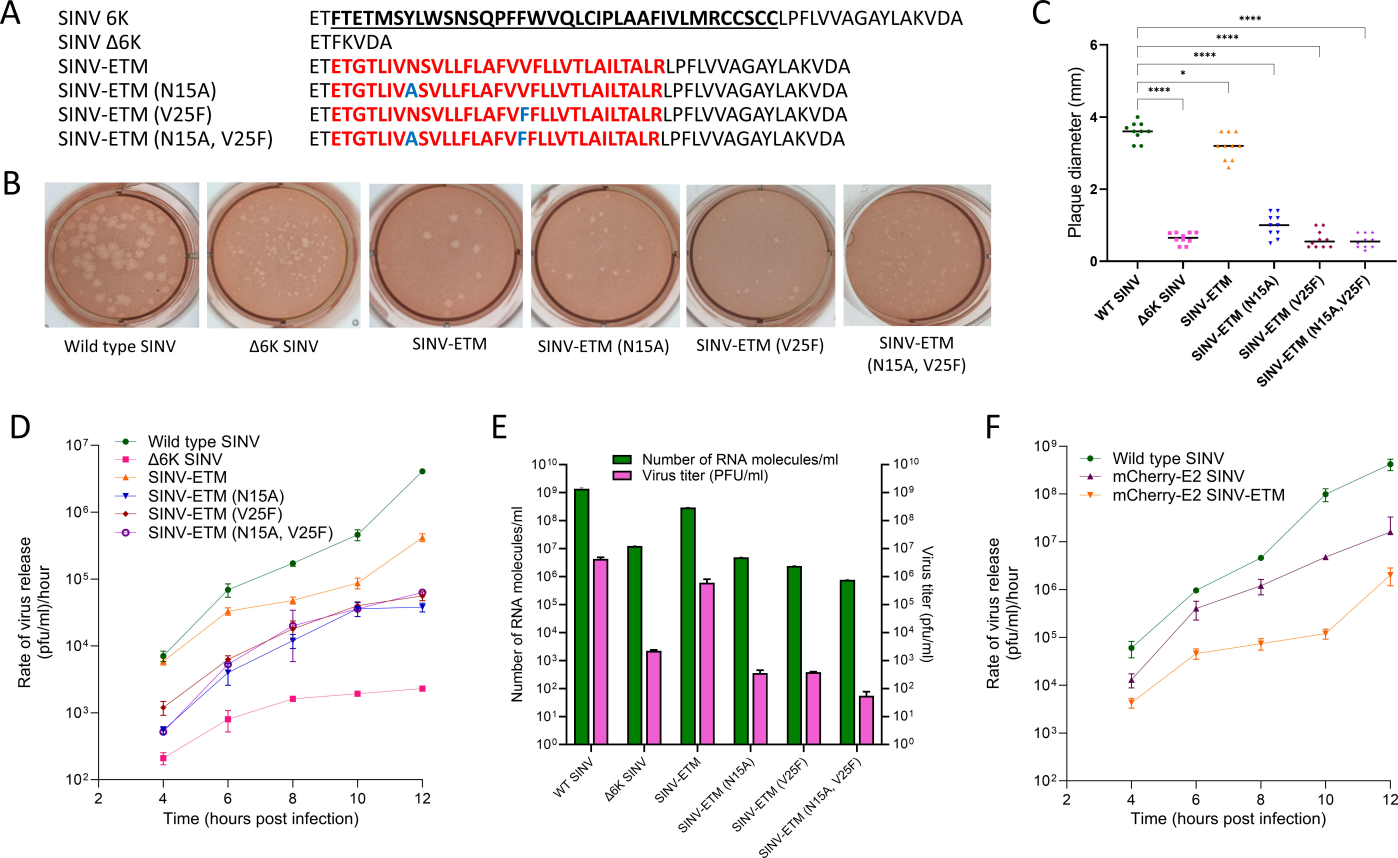
Generation and growth kinetics of SINV 6K ion-channel chimeras. (**A**) The amino acid sequences of 6K and 6K mutant chimeras—WT SINV, ∆6K SINV, SINV-ETM, SINV-ETM (N15A), SINV-ETM (V25F), and SINV-ETM (N15A, V25F). The ETM sequence is shown in red with channel-inactivating mutations in blue. (**B**) Plaque morphologies of viruses mentioned in (**A**) grown in BHK-21 cells for 2 days after electroporation. (**C**) Differences in mean plaque diameters of 10 randomly selected plaques for every mutant virus were compared to WT SINV using GraphPad Prism software. Dunnett’s multiple comparisons test as part of one-way ANOVA was used to determine statistical significance with a 95% confidence interval. *P < 0.05, **P < 0.01, ***P < 0.001, ****P < 0.0001 and ns-not significant. (**D**) One-step growth curves of wild-type and channel mutant viruses over 12 hours of infection in BHK-21 cells. Cells were infected at an MOI of 1, and virus-containing media were harvested at given time points to indicate the rate of virus release per hour. Data are representative of two independent experiments. (**E**) Comparison of viral titers and the total number of viral RNA genomes produced by wild-type and mutant viruses 12 hours after electroporation with *in vitro* transcribed RNA. The experiment was performed in triplicate, and error bars represent the standard deviation (SD). (**F**) One-step growth curves of wild-type and mCherry-tagged SINV and SINV-ETM chimera at an MOI of 2. Data are representative of two independent experiments.

A SINV 6K channel chimera replacing the first transmembrane helix of 6K with the TM domain of SARS-CoV-2 E (SINV-ETM), residues 8–38 (40), was designed as shown in Fig. 1A. The signalase cleavage sites flanking 6K in the SINV genome and the frameshift site needed for TF production were kept intact. By replacing only the first transmembrane helix, the wild-type topology of 6K is maintained in the chimera. We used ∆6K SINV, an ion-channel-deficient SINV mutant previously described in Elmasri et al. (32), as a control to assess the ion-channel activity of the SINV-ETM chimera (Fig. 1A). That study showed that ∆6K SINV had a significant reduction in plaque size and an ~4 log reduction in viral titer compared to wild-type virus after 12 hours of infection at a multiplicity of infection (MOI) of 2.0 in BHK-21 cells. Elmasri and colleagues further showed that these defects were rescued to varying degrees after introducing ion-channel domains of viroporins of enveloped viruses such as human immunodeficiency virus type-1 (HIV-1) Vpu, hepatitis C virus (HCV) P7 and IAV-M2 (32). In the SINV-ETM chimera, we observed a visible and statistically significant increase in plaque size compared to ∆6K SINV (Fig. 1B and C). Following the viral rescue, we examined the growth kinetics of the chimera using one-step and continuous growth curve analyses at MOIs of 1.0 and 0.1 in BHK-21 cells (Fig. 1D; Fig. S1). We observed a 2.5-log increase in viral titer after 12 hours compared to ∆6K SINV. Next, we performed qRT-PCR to quantify the number of viral RNA genomes in our virus samples 12 hours post-electroporation with *in vitro* transcribed RNA. These values were divided by the viral titers to determine specific infectivity (SI) ratios (Fig. 1E). While ∆6K SINV had a ~17-fold higher SI compared to WT SINV, indicative of a large population of non-infectious viruses in the former, SINV-ETM chimera had a similar SI ratio compared to wild-type virus (Table 1). This further showed that the ETM channel chimera can substantially rescue defects shown in ∆6K SINV, which led to the formation of non-infectious viruses.

**TABLE 1:** Specific infectivity (SI) ratios of WT SINV and mutant viruses in BHK cells (12 hours post-electroporation)

Virus	SI (genomes/titer)
WT SINV	324
Δ6K SINV	5,587
SINV-ETM	492
SINV-ETM (N15A)	13,759
SINV-ETM (V25F)	6,344
SINV-ETM (N15A, V25F)	13,995

To further test whether the rescue in viral titer and plaque size in the ETM chimera is due to a functional ion channel, channel-inactivating mutations were introduced in the ETM sequence: N15A, V25F and the double mutant N15A, V25F (Fig. 1A). Previous studies done in SARS-CoV have shown that these single substitutions completely abolish channel activity of E protein in lipid bilayers (34, 48) while the double substitution has reduced conductance (34). Revertants of these single viral mutants obtained from studies in mice and Vero E6 cells had similar conductance as the wild-type protein when tested in lipid membranes (49). Since SARS-CoV and SARS-CoV-2 E proteins have 100% sequence identity in the TM domain, similar results can be expected for the latter (33, 37). We observed reduced plaque sizes for these mutants in our chimera similar to ∆6K SINV (Fig. 1B) and a ~ 0.5–1 log lower titer than SINV-ETM in one-step growth curve analysis (Fig. 1D). The SI ratios of these mutants were also similar to ∆6K SINV in the case of V25F and higher in the case of N15A and the double substitution mutant. Thus, the ETM chimera can cis-complement the function of the 6K ion channel domain and rescue defects in SINV growth, which can be hampered by introducing mutations that affect the ETM channel activity.

### Channel inhibitors such as amantadine and amiloride reduce viral infection in SINV 6K and SINV-ETM chimera

**Fig 2:**
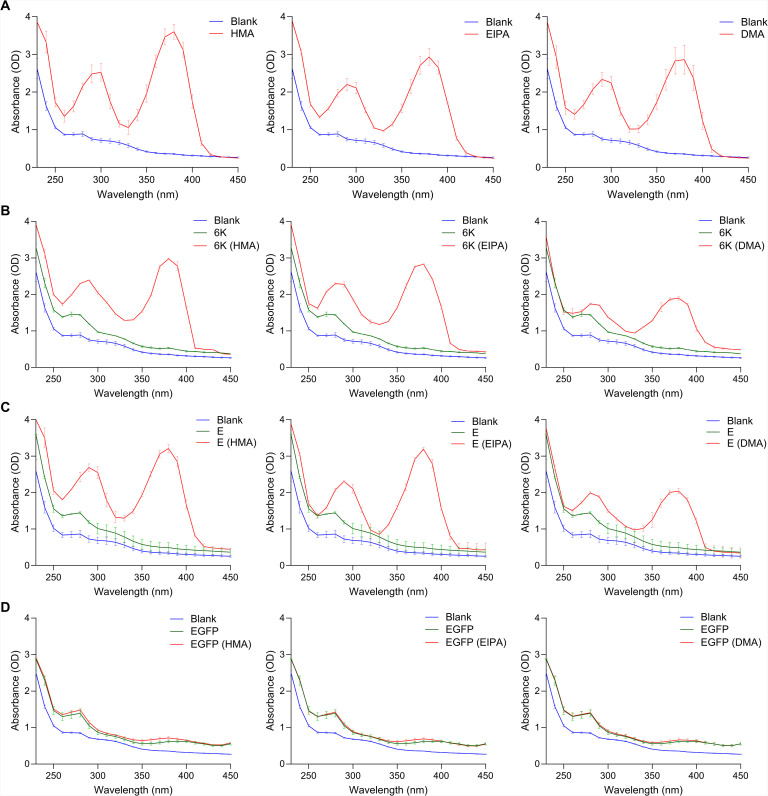
SINV 6K and SARS-CoV-2 E proteins bind to amiloride derivatives. UV-Vis spectra of (**A**) HMA, EIPA, and DMA. UV-Vis spectra of compounds bound to (**B**) SINV 6K, (**C**) SARS-CoV-2 E, and (**D**) EGFP proteins. Blank refers to the absorbance of the dialysis buffer after unbound compounds have been removed from each sample. The data shown represent two independent experiments.

Amantadine and amilorides such as hexamethylene amiloride (HMA) are known inhibitors of channel activity of viroporins (19, 33, 34, 40, 44). To establish our chimeric system as a tool to study SARS-CoV-2 E-channel activity and its inhibition, we tested these compounds against the ETM channel in the SINV chimeric background and compared the results to previous studies (40). We first tested the binding of three amiloride derivatives—HMA, EIPA, and DMA—with purified SINV 6K, full-length SARS-CoV-2 E, and E protein with channel substitutions in DDM micelles expressed in bacteria (Fig. S2A through D) using UV-Vis absorption spectroscopy. To ensure the specificity of binding interaction, purified EGFP protein was used as a negative control (Fig. S2E and F). Amantadine does not have a characteristic absorption in the UV-Vis spectrum and hence was left out of the experiment. HMA, EIPA, and DMA have characteristic absorption peaks around 290 and 380 nm (Fig. 2A). The proteins were incubated with 3–4× molar excess of the compounds for 1 hour at 4°C and dialyzed overnight in a 3 kDa cut bag. Upon dialysis, the unbound compound was removed while the fraction of the compounds bound to the proteins was retained in the dialysis bag. Both 6K (Fig. 2B) and E (Fig. 2C) proteins bind to the compounds, although to a lesser extent in the case of DMA, as indicated by a lower OD value around 290 and 380 nm for the same amount of protein. The EGFP control does not show binding to any of the compounds (Fig. 2D). Since N15A and V25F mutations in SARS-CoV E are known to affect oligomeric states of the protein (34, 38), we also tested the single and double substitution mutants for their ability to bind the compounds. All three mutants showed binding, although to a slightly lesser degree than the wild-type protein (Fig. S3). This is expected considering the mutant proteins also show oligomeric bands near 25 and 15 kDa on SDS-PAGE (Fig. S2C).

For ease in visualization and quantification of the effect of channel inhibitors on the chimeric viruses, an mCherry tag was inserted at the N-terminus of SINV E2 as described in Jose et al. (45) in all constructs mentioned in Fig. 1A. One-step growth curve analysis of mCherry-tagged WT SINV and SINV-ETM chimera was performed (Fig. 1F). As expected, based on Fig. 1D, ~1-log reduction in titer was observed for the tagged SINV-ETM chimera compared to tagged wild-type SINV. Using a calorimetric cell-proliferation assay, cytotoxicity profiles of HMA, EIPA, DMA, and amantadine were determined for BHK-21 cells (Table 2). HMA had the highest cytotoxicity followed by EIPA and DMA while amantadine had the lowest.

**TABLE 2:** CC_50_ values of inhibitors in BHK cells as shown in graphs of Fig. 3A for 12 hour incubation and Fig. S4 for 24 hour incubation

Inhibitor	12 hour incubation	24 hour incubation
HMA	66.8 µM	46.03 µM
EIPA	>100 µM	93.93 µM
DMA	>100 µM	>100 µM
Amantadine	2.24 mM	1.94 mM

**Fig 3:**
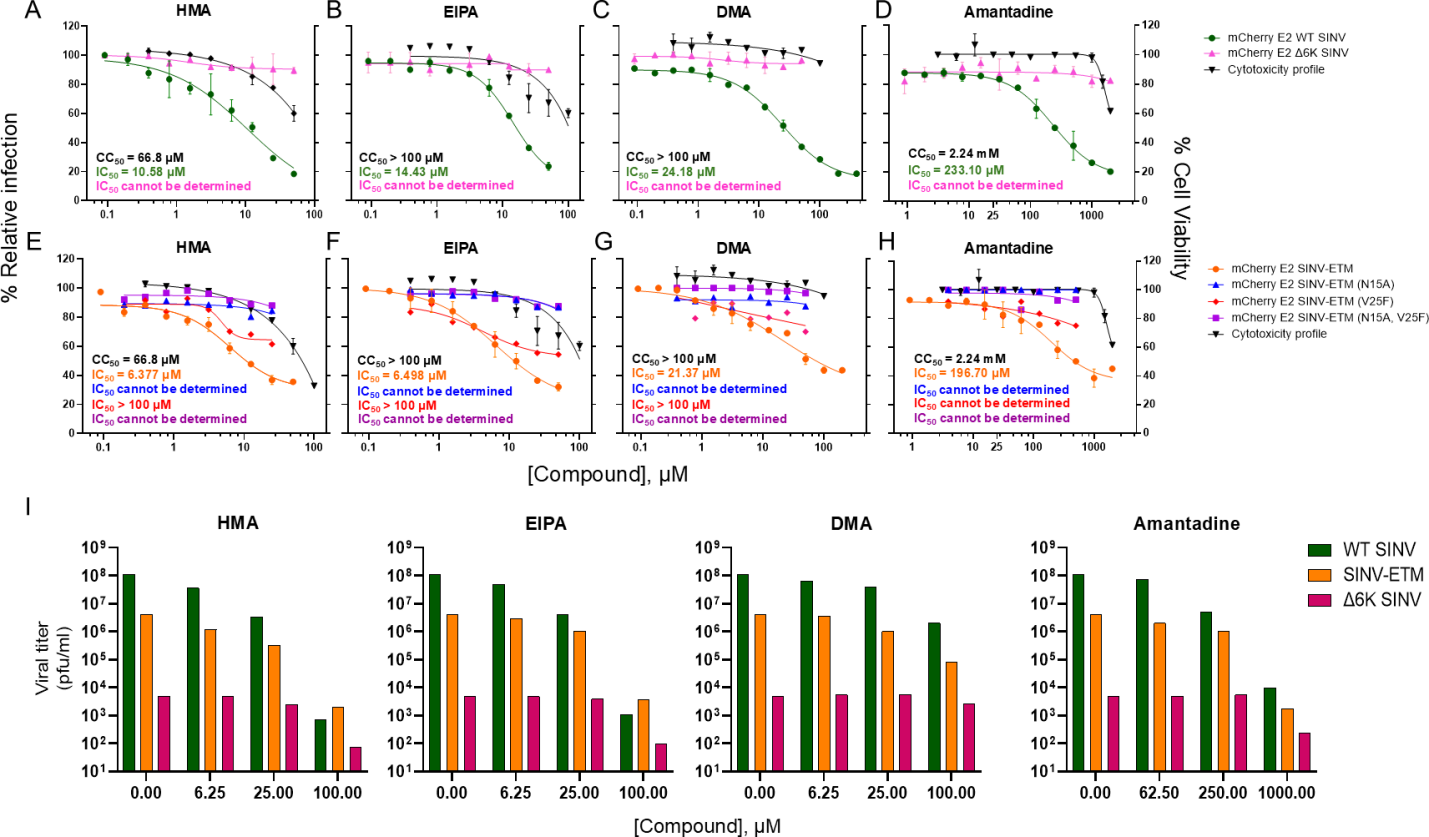
Inhibition of SINV and SINV-ETM mutant virus infection by amilorides and amantadine in BHK cells at MOI of 1.0 and 12 h incubation. BHK cells were treated with indicated concentrations of (**A, E**) HMA, (**B, F**) EIPA, (**C, G**) DMA, and (**D, H**) amantadine and infected with (**A–D**) mCherry E2 WT SINV (green), mCherry E2 Δ6K SINV (pink) and (**E–H**) mCherry E2 SINV-ETM (orange), ETM (N15A) (blue), ETM (V25F) (red), ETM (N15A, V25F) (purple) viruses at an MOI of 1.0 for 12 hours. (**I**) BHK-21 cells were infected with untagged WT SINV, ∆6K SINV, and SINV-ETM chimera at an MOI of 1.0 treated with indicated concentrations of inhibitors and plaqued after 12 hours.

Based on the CC_50_ values, concentration ranges for the compounds were determined and tested for inhibition of viral growth with mCherry-tagged viruses. BHK-21 cells were infected with an MOI of 1.0 of wild-type SINV and mutant viruses in the presence of a compound or DMSO control (Fig. 3). After 12 hours, the mCherry fluorescence signal in each compound-treated sample was used to measure the percent relative infection to calculate IC_50_ values (Fig. 3A through H). All four compounds showed concentration-dependent inhibition against SINV (Fig. 3A through D) and SINV-ETM chimera (Fig. 3E through H) but had little to no effect on ∆6K SINV (Fig. 3A through D). HMA and EIPA showed higher levels of inhibition compared to DMA for the chimera, a result similar to a previous study done on the authentic SARS-CoV-2 virus in Vero E6 cells (40) while amantadine had the highest IC_50_ concentration. We also observe the same order of antiviral activity (HMA >EIPA >> DMA >>>Amantadine) for SINV, further validating that 6K and SARS-CoV-2 E ion channels function in a similar fashion in SINV. The assay was also performed for low MOI virus (MOI = 0.1) treated with inhibitors for 24 hours and the same trend was observed (Fig. S4). The compounds show a lesser degree of inhibition against SINV-ETM channel mutants compared to ETM as seen in Fig. 3E through H. This result is expected since these mutants do not completely abolish channel activity and would not behave similarly to ∆6K SINV.

**Fig 4:**
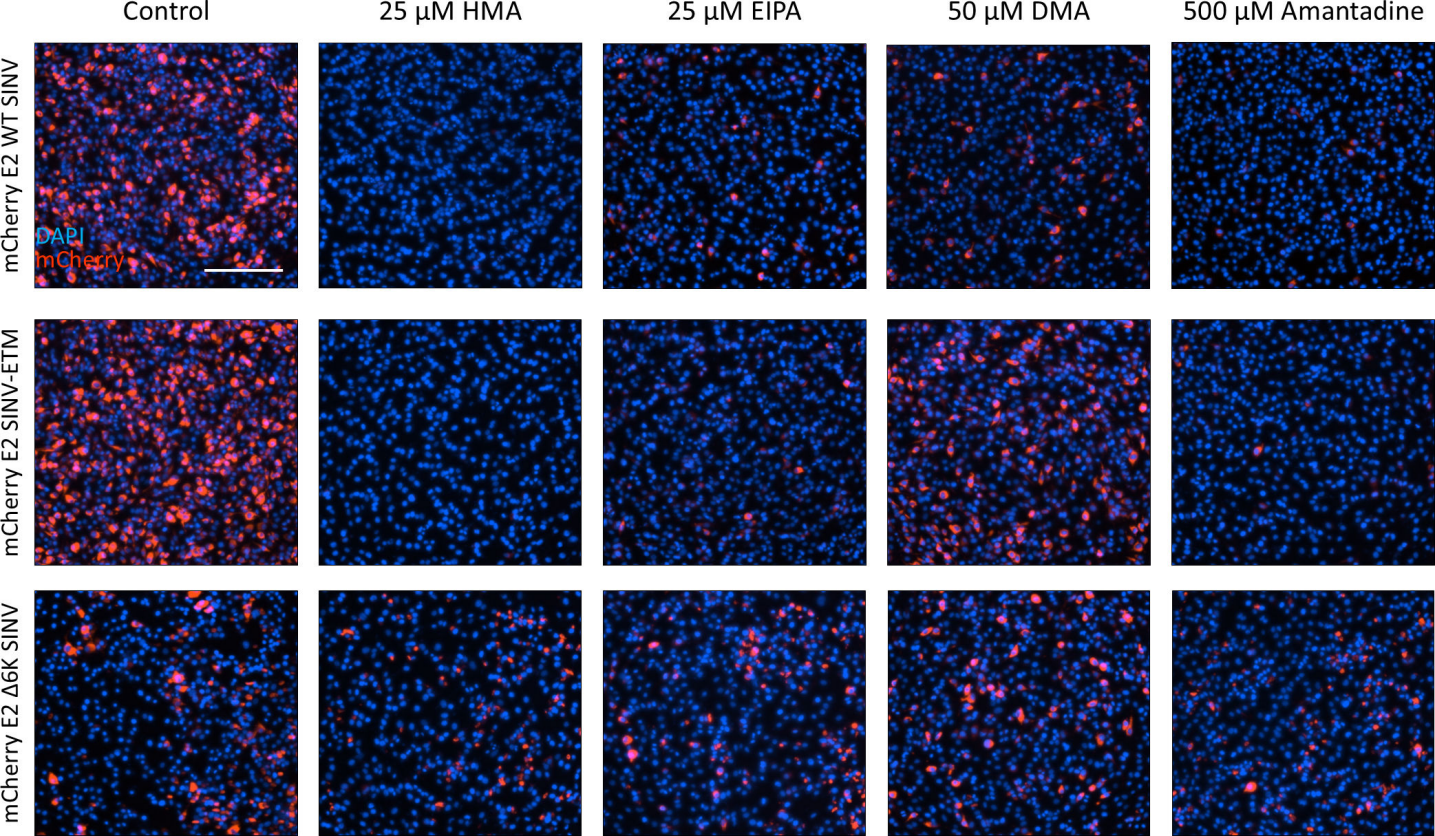
Inhibition of viral infection with channel inhibitors in BHK-21 cells infected at MOI 1.0. Cells infected with mCherry-tagged viruses were treated with 0.1% DMSO (control) and compound inhibitors at the indicated concentrations for 12 hours and stained for DAPI nuclear stain (blue). Cells were imaged at 20× magnification on Cytation 7 using DAPI and TRITC (red) channels. Scale bar = 200 µm

To test the reliability of the above method of determining inhibition by measuring the mCherry signal, plaque assays were performed with the untagged viruses—WT SINV, ∆6K SINV, and SINV-ETM chimera—treated with the inhibitors in BHK-21 cells and harvested after 12 hours. Titers were compared to DMSO-treated virus and plotted as percentages (Fig. 3I). The results follow the same inhibitory trend as seen in Fig. 3A through H. The inhibition of growth can also be visualized in tagged viruses when treated with concentrations of compounds greater than the IC_50_ concentration (Fig. 4).

### Time of inhibitor addition assay reveals a functional role for an ion channel during the early stages of the SINV life cycle

Both alphavirus 6K and SARS-CoV-2 E proteins are known to play important roles in the later stages of their respective virus life cycles, although the exact mechanisms for their action are not known. 6K has long been known to be involved in the budding process (31) and was recently shown to affect glycoprotein trafficking and CPV-II formation through its ion channel activity (32). SARS-CoV-2 E protein localizes to the ER-Golgi intermediate compartment (ERGIC), the site of coronavirus assembly (50), and is known to cause membrane curvature and disruption of calcium homeostasis via its ion channel (51). Therefore, it is likely that the channel inhibitors affect the assembly/egress stage of SINV and SINV-ETM infection. We performed an egress inhibition assay to test the effect of the compounds on late-stage infection. Infected BHK-21 cells were treated with the compounds after 8 hours of infection, and the supernatants were harvested at 12 hours post-infection (hpi). These supernatants were used to perform a plaque assay to quantify the amount of infectious virus produced when the compounds are added at later stages. The viral titers of WT SINV and SINV-ETM are reduced by approximately 0.4–1.0 logs in the presence of the compounds, while little to no change is observed in the viral titer of ∆6K SINV (Fig. S5). This demonstrates the expected effect of the channel inhibitors on viral egress.

**Fig 5:**
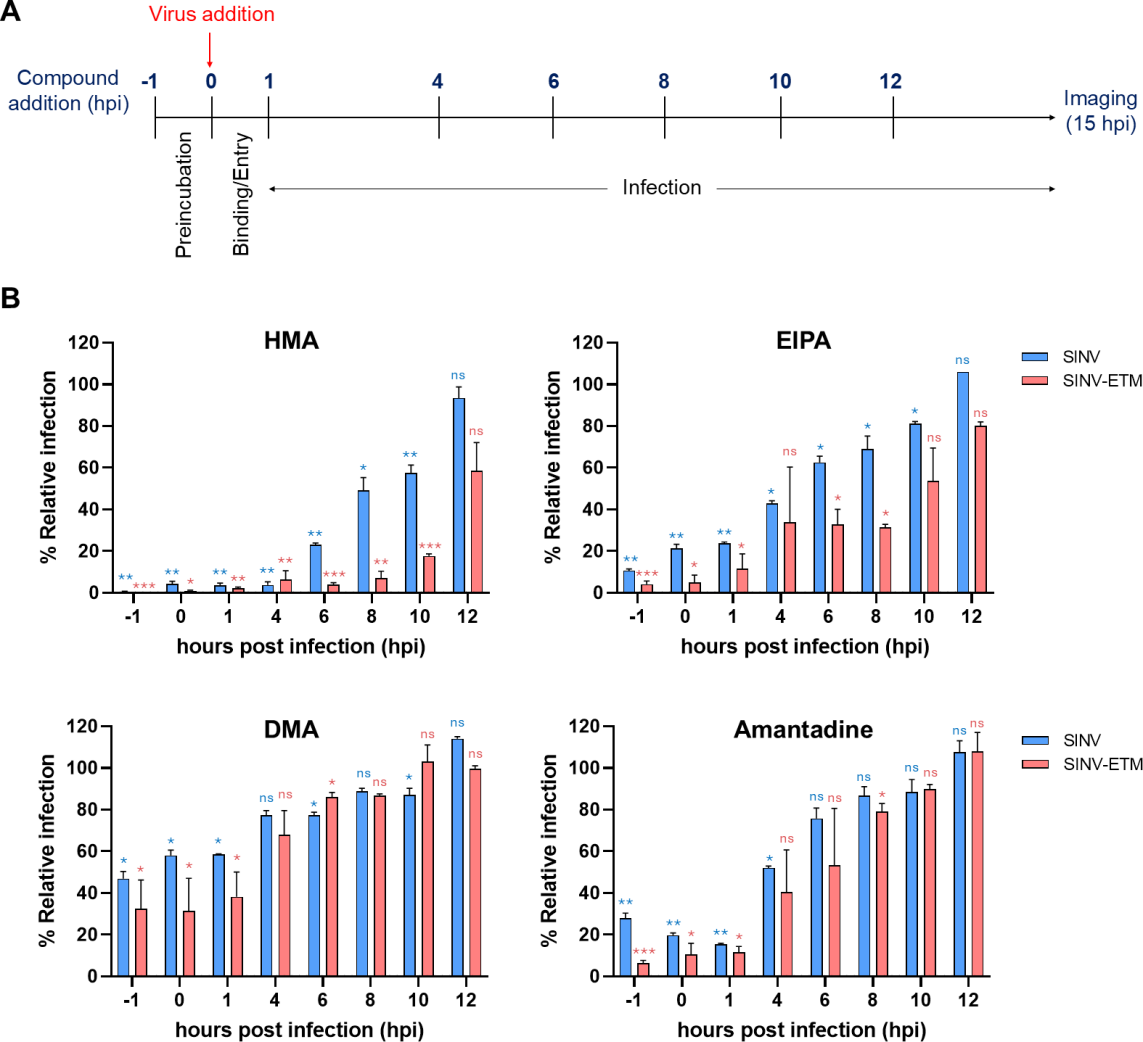
Time of inhibitor addition assay at high MOI. (**A**) Schematic representation of the time of addition assay protocol. (**B**) Time of addition assay in BHK-21 cells with channel inhibitors. Cells were infected with either mCherry-E2-tagged SINV or SINV-ETM virus at t = 0 with a high MOI of 10. 25 µM HMA, 25 µM EIPA, 50 µM DMA, and 500 µM Amantadine were added to the cells at the indicated time points. Cells were fixed and imaged at 15 hpi at 4× magnification. The fluorescence intensity signal (TRITC channel) was measured for each sample relative to the DMSO-control signal and plotted for graphical representation. Error bars represent the standard error of the mean (SEM) of two independent experiments. Statistical significance was determined for each time point between inhibitor-treated and DMSO control values by two-tailed unpaired t-test with a 95% confidence interval. *P < 0.05, **P < 0.01, ***P < 0.001, ****P < 0.0001 and ns-not significant.

To further investigate what stage of viral infection is affected by the compounds used in this study, a time of addition assay was performed. Figure 5A shows a schematic of the different time points of the addition of compounds. Compounds were used at ~2–3 times the IC_50_ concentration. First, we tested mCherry-tagged SINV and SINV-ETM infection at a low MOI of 0.1 (asynchronous infection) to capture changes in overall infection. For both viruses, we see smaller foci of mCherry-labelled cells compared to the control with 50 µM of DMA up to 6–8 hpi, beyond which the effect is lost (Fig. S6B and C). This is expected since channel activity is needed for budding and spreading after the first round of infection. 8 hpi is also the time point when the expression of mCherry-tagged E2 glycoprotein is highest on the cell surface (45). A similar result is seen in the case of 500 µM amantadine treatment of WT SINV-infected cells (Fig. S6B). For SINV-ETM, however, little to no infection is seen at early time points (−1, 0, 1, and 4 hpi) upon amantadine treatment (Fig. S6C). Amantadine, a lysosomotropic weak base, has been shown to reduce alphaviral entry by increasing endosomal pH (52). In addition, 25 µM HMA and EIPA treatment resulted in nearly complete inhibition of infection at earlier time points for both viruses, with HMA being more potent than EIPA (Fig. S6B and C).

To test whether ion-channel activity of 6K and ETM is needed for entry in SINV infection, we performed the assay with a high MOI of 10 (synchronous infection) (Fig. 5, Fig. S6D and E). The results are even more striking at an MOI of 10, where the addition of the channel inhibitors leads to lower infection levels when added at earlier time points (Fig. S6D and E). All four compounds show a statistically significant reduction in viral infection, with HMA, EIPA, and amantadine being more potent than DMA at the chosen concentrations (Fig. 5B). For analyzing the results at ~4–6 hpi, it is important to note that CPV-IIs carrying E1/E2 glycoproteins originate around 4 hpi in WT SINV and need a functionally active ion channel for biogenesis (45). A delay in mCherry-E2 trafficking due to channel inhibition should, however, not result in loss of fluorescence signal at 15 hpi. This also does not explain the results seen at −1, 0, and 1 hour time points (Fig. 5B). It is also possible that higher concentrations of DMA and amantadine might have yielded results similar to HMA and EIPA. In a separate experiment, we also looked at the effect of preincubation with HMA on viral entry of WT SINV and ∆6K SINV using a plaque number reduction assay (Fig. S7). Preincubation with HMA for 1 hour resulted in a 0.2–0.3 log reduction in the viral titer of WT SINV but not of ∆6K SINV. The time of addition assay and the plaque number reduction assay, along with the huge differences in SI ratios seen in Fig. 1E and Table 1, could be indicative of a channel role in entry.

## DISCUSSION

The channel activity of the coronavirus E protein has been implicated in the assembly and release of virus particles via the formation of membrane curvature and disruption of normal cellular functions (33, 49, 51). More recent studies with SARS-CoV-2 have shown that the E protein is needed for overall viral fitness and pathogenesis, including triggering of robust inflammatory immune responses and induction of cell death (39). It has also been shown that E and M (membrane) proteins of SARS-CoV and SARS-CoV-2 are the minimum requirements for a VLP (virus-like particle) system (53, 54), strongly suggesting that the two proteins interact during assembly (55). However, conflicting reports exist suggesting that while the inclusion of E enhances the VLP production, the M and N proteins represent the minimum VLP requirements (56, 57). Another recent study has shown that SARS-CoV-2 uses a lysosome-based egress pathway instead of the conventional biosynthetic secretory pathway (58). The ability of the E protein to alter the pH of intracellular compartments and disrupt calcium homeostasis could be the basis for lysosomal deacidification needed in this pathway. Thus, E protein has multiple functions in the viral life cycle, and although it is not essential for particle production, it can greatly affect viral growth as seen in various channel inhibition studies (37, 40, 44). Antivirals that target the channel activity of E have the potential to assist in combating COVID-19 infection.

In this study, we propose a fluorescently tagged SINV chimeric system for screening channel inhibitors against the ion-channel activity of SARS-CoV-2 E protein in a BSL-2 environment. Our method has proven useful in testing known inhibitors and has the potential to be used for testing novel inhibitors in a high-throughput manner without the need for a BSL-3 laboratory. It is a faster, safer, and cheaper method to screen compound libraries before testing in an authentic SARS background. We have exploited the structural and functional similarities between viroporins of enveloped viruses to generate a SINV-ETM chimera and show that the ion channel of E protein can, to a large extent, rescue growth defects that occur due to deletion of the SINV 6K as shown in Fig. 1. To ensure that the rescue is due to the presence of a functional ion channel, we introduced channel-disrupting mutations in the ETM sequence that reverse the rescue, although not to the extent of Δ6K SINV.

Next, we tested our chimera against known channel inhibitors of E to further establish the reliability of the system as a compound screening tool. First, we tested the purified 6K and E proteins for binding with these inhibitors *in vitro* (Fig. 2), and then proceeded to perform inhibition studies with these compounds in the chimeric system to calculate IC_50_ values. For faster evaluation, we used the total mCherry signal produced compared to DMSO controls as a read-out for change in overall infection. We optimized our protocol on an iD5 spectrophotometer for quantification, which was validated using plaque assays and qualitative analysis of imaging data shown in Fig. 3 and 4. Our results were similar to previously reported results where HMA and EIPA performed better than DMA and amantadine but had higher cytotoxicity in Vero E6 cells (40, 44).

Of the two residues modified in ETM to disrupt channel activity, N15A was more resistant to the effect of the inhibitors compared to V25F (Fig. 3E through H). The purification and gel analysis profiles of the mutants, however, are identical (Fig. S2). Previous studies based on the NMR structure of E have shown that compounds such as HMA and amantadine interact with the N-terminus of the protein (37, 44). The N15 residue was shown to be very close to the binding sites of these compounds, and its substitution to alanine modified the channel conformation needed for binding. This could explain why the N15A mutant chimera is almost unaffected by the addition of the compounds shown in Fig. 3.

Viroporins are known to assist in the later stages of infection, and the addition of channel inhibitors blocks viral egress in both WT SINV and SINV-ETM chimera as expected (Fig. S5). To further narrow down the possible stage of the viral life cycle affected by these inhibitors and gain insight into their possible modes of action, we performed a time of addition assay in BHK-21 cells with the tagged WT SINV and SINV-ETM chimera. Surprisingly, even at a high MOI of 10, the addition of the compounds at earlier time points greatly reduced the level of infection, with HMA being the most potent at the concentrations tested (Fig. 5, Fig. S6D and E). This hints toward a possible role of the ion channels in SINV entry and/or replication. Further studies are needed to validate whether ion-channel activity assists in the entry/uncoating of the SINV particles. Ion channels such as IAV-M2 have been known to assist in uncoating of the virus particles while viroporins of some picornaviruses and rotaviruses are involved in replication as well (17). Therefore, it is possible that SINV 6K/TF is also involved in entry as it is present in sub-stoichiometric amounts in budding virions (30, 59). This would explain the vast differences in SI ratios of WT SINV and Δ6K SINV in Fig. 1E and the decrease in SINV titer upon preincubation with HMA in Fig. S7. The E ion channel is involved in post-entry stages of SARS-CoV-2 infection, and a role for the channel in entry has not been established. Future studies are needed to independently investigate whether the E channel is involved in SARS-CoV-2 entry. Studies are also needed to determine the functional oligomeric state(s) of 6K to understand the mechanism of action of its ion channel and how channels of other proteins can complement those roles.

SINV and SARS-CoV-2 utilize distinct pathways for viral exit. The assembly of SARS-CoV-2 virions occurs in the ERGIC, and the newly formed particles are released through the secretory pathway (60, 61). In SINV, assembly and budding occur at the plasma membrane (62). While 6K, similar to E, localizes to the ER and Golgi compartments and is largely absent from the plasma membrane, their intracellular distribution during their respective life cycles may not fully overlap. SINV also encodes the TF variant of 6K which can traffic to the plasma membrane for assembly into budding particles (20). This presents a limitation of the SINV chimeric system in evaluating the effects of E-channel function, particularly roles that are poorly characterized or unknown in the authentic SARS-CoV-2 system, such as a potential role in viral entry. Nevertheless, the SINV chimeric system provides a useful and convenient tool to study the inhibition of these channels and can be exploited for the initial screening of compound libraries in an effort to find life-saving antivirals against high-containment coronaviruses.

## Data Availability

Data supporting the findings of this study are available within the article and its supplemental material.

## SUPPLEMENTAL MATERIAL

Supplemental material jvi.02252-24-s0001.docx Figures S1 to S7.

ASM does not own the copyrights to Supplemental Material that may be linked to, or accessed through, an article. The authors have granted ASM a non-exclusive, world-wide license to publish the Supplemental Material files. Please contact the corresponding author directly for reuse.
