# Supplementary material for: A BSL-2 chimeric system designed to screen SARS-CoV-2 E protein ion channel inhibitors
Source: J Virol. 2025 Apr 30;99(5):e02252-24. doi: 10.1128/jvi.02252-24 (PMC12090776; doi:10.1128/jvi.02252-24)
Supplement: Supplemental material — Figures S1 to S7. [file jvi.02252-24-s0001.docx]

**Supplementary Data**


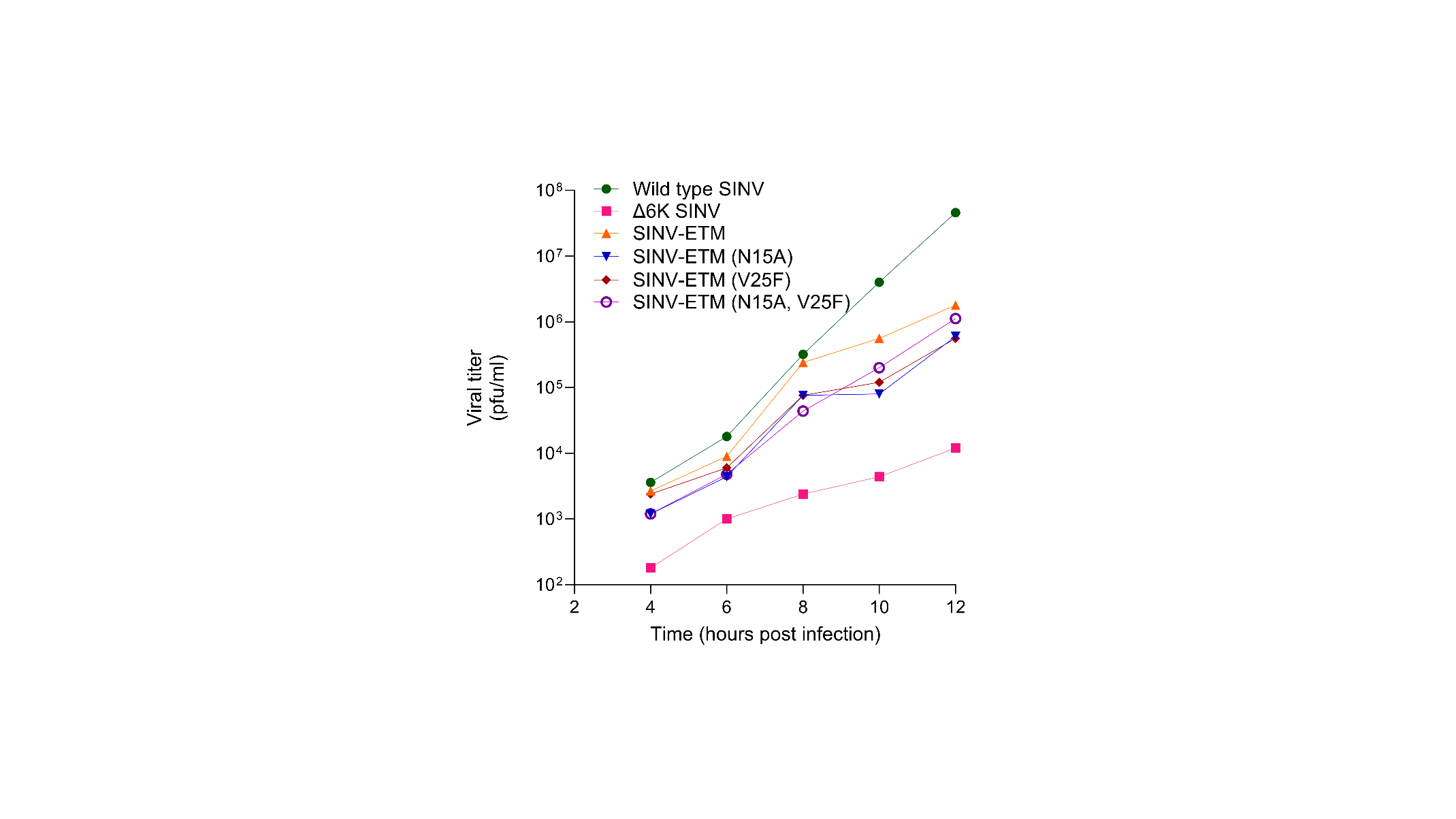


**Figure S1.** **Continuous growth curve analysis of WT and channel mutant viruses at MOI of 0.1.** Cells were infected with virus samples at an MOI of 0.1 and virus-containing media was harvested at indicated time points. The experiment was performed once.


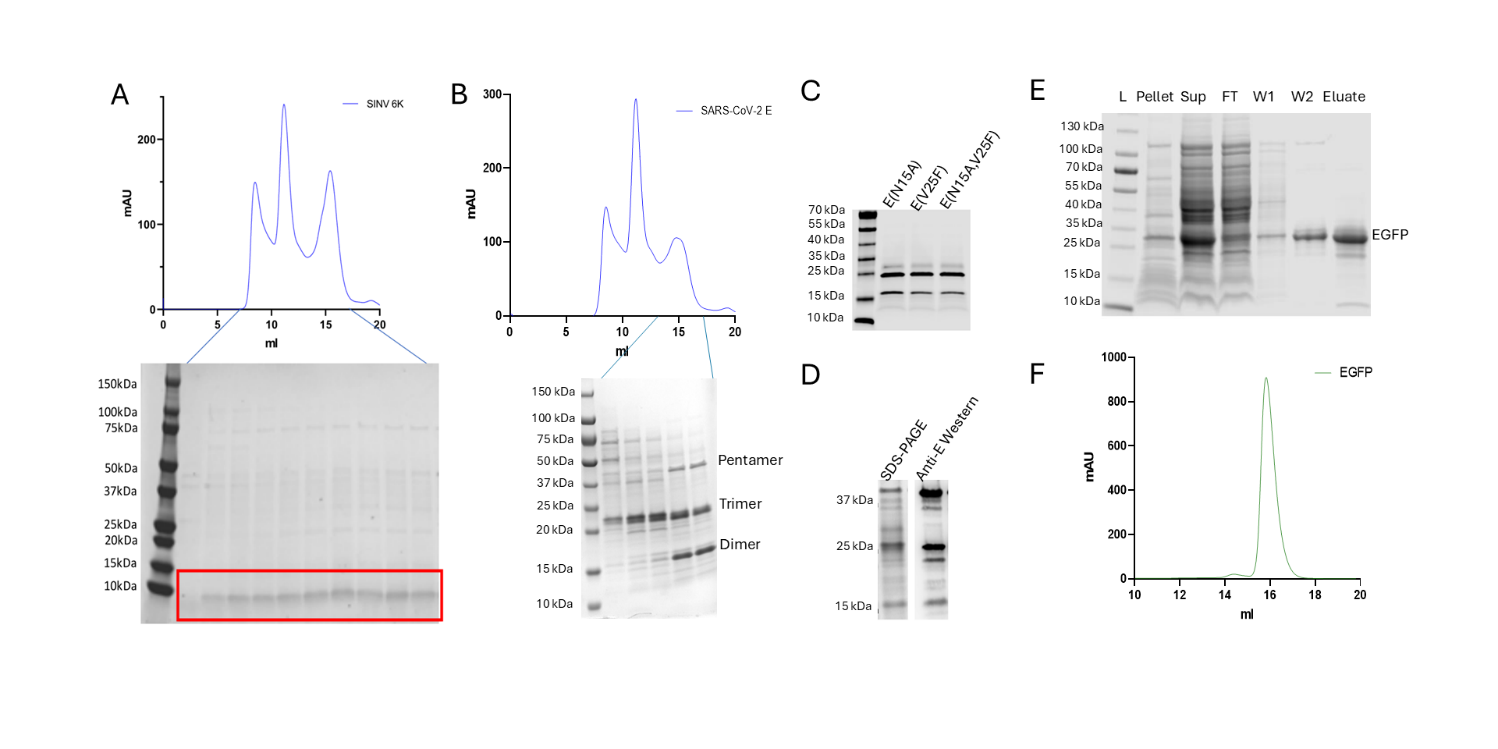


**Figure S2. Protein purification data.** (A and B) Size exclusion chromatography (SEC) (top) with SDS-PAGE gel profiles of the SEC fractions (bottom) of SINV 6K and SARS-CoV-2 E proteins expressed in bacteria and purified in DDM micelles. (A) 6K under reducing conditions gives a band below 10kDa marker shown in red at the expected band size for the monomer form of the protein (6kDa). (B) E protein forms various oligomers (corresponding to a pentamer, a trimer and a dimer) that are SDS-resistant, similar to previously reported purification profiles^58,59^. These oligomers remain intact under reducing conditions since ETM domain has no cysteine residues. (C) SDS-PAGE profile of purified E channel mutants shows oligomeric states similar to wild-type E, however the pentamer form is not visible. (D) SDS-PAGE and western blot profiles of purified SARS-CoV-2 E protein using E antibody. The different SDS-PAGE bands corresponding to different oligomeric states of the protein are detected by SARS-CoV-2 E antibody. (E) Ni-NTA purification profile of His-tagged EGFP expressed in bacterial cells. (F) SEC profile of EGFP protein shows a single Gaussian peak corresponding to the purified protein.

mAU, milli-absorbance units; WB, western blot; L, ladder; sup, supernatant; FT, flow-through; W1, first wash; W2, second wash.


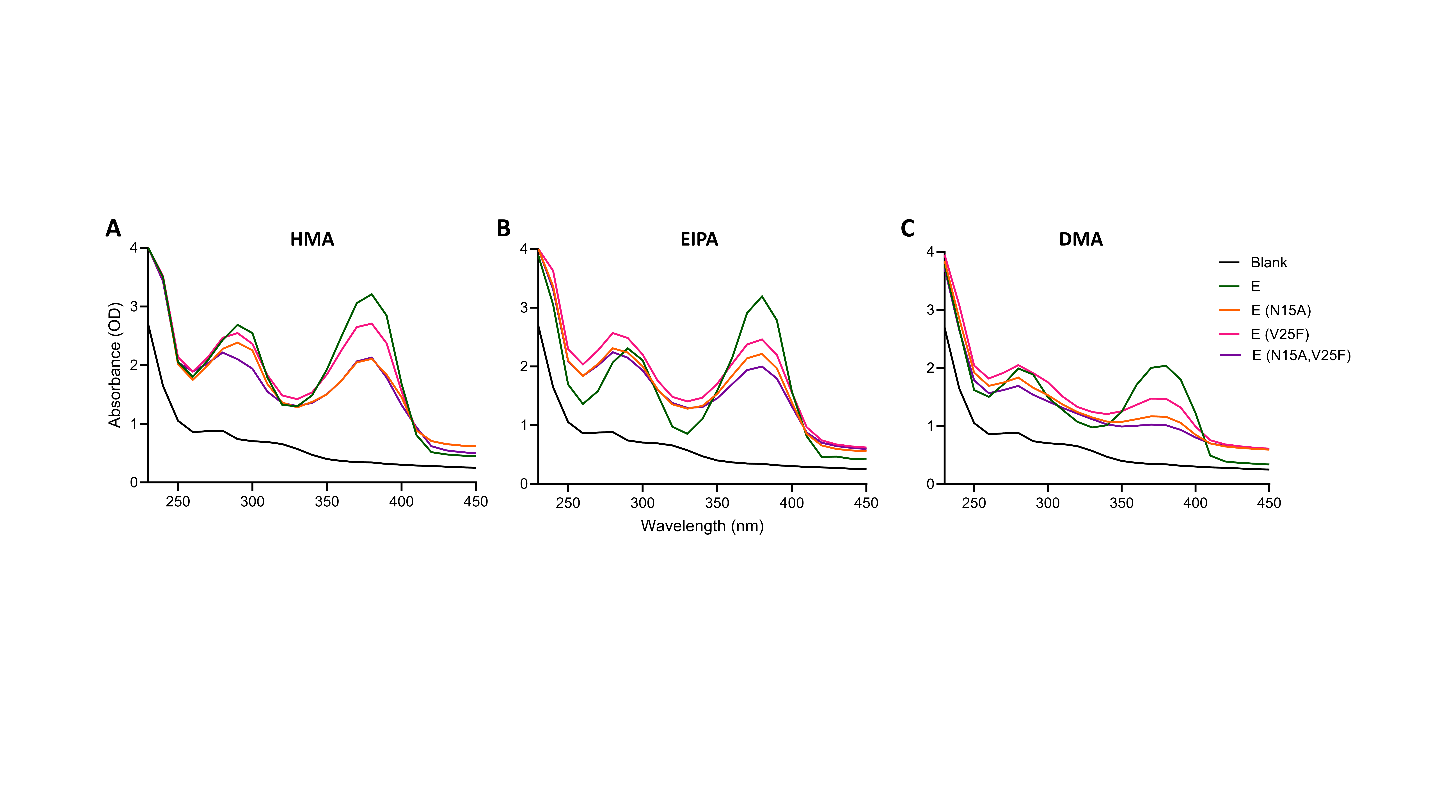


**Figure S3. SARS-CoV-2 E channel mutant proteins show decreased binding to amiloride derivatives than wild-type E.** UV-Vis spectra of wild-type E and its mutants E(N15A), E(V25F) and the double mutant bound to (A) HMA, (B) EIPA, and (C) DMA. Blank refers to the absorbance of the dialysis buffer after unbound compounds have been removed from each sample. Data shown are the average results of two independent experiments.


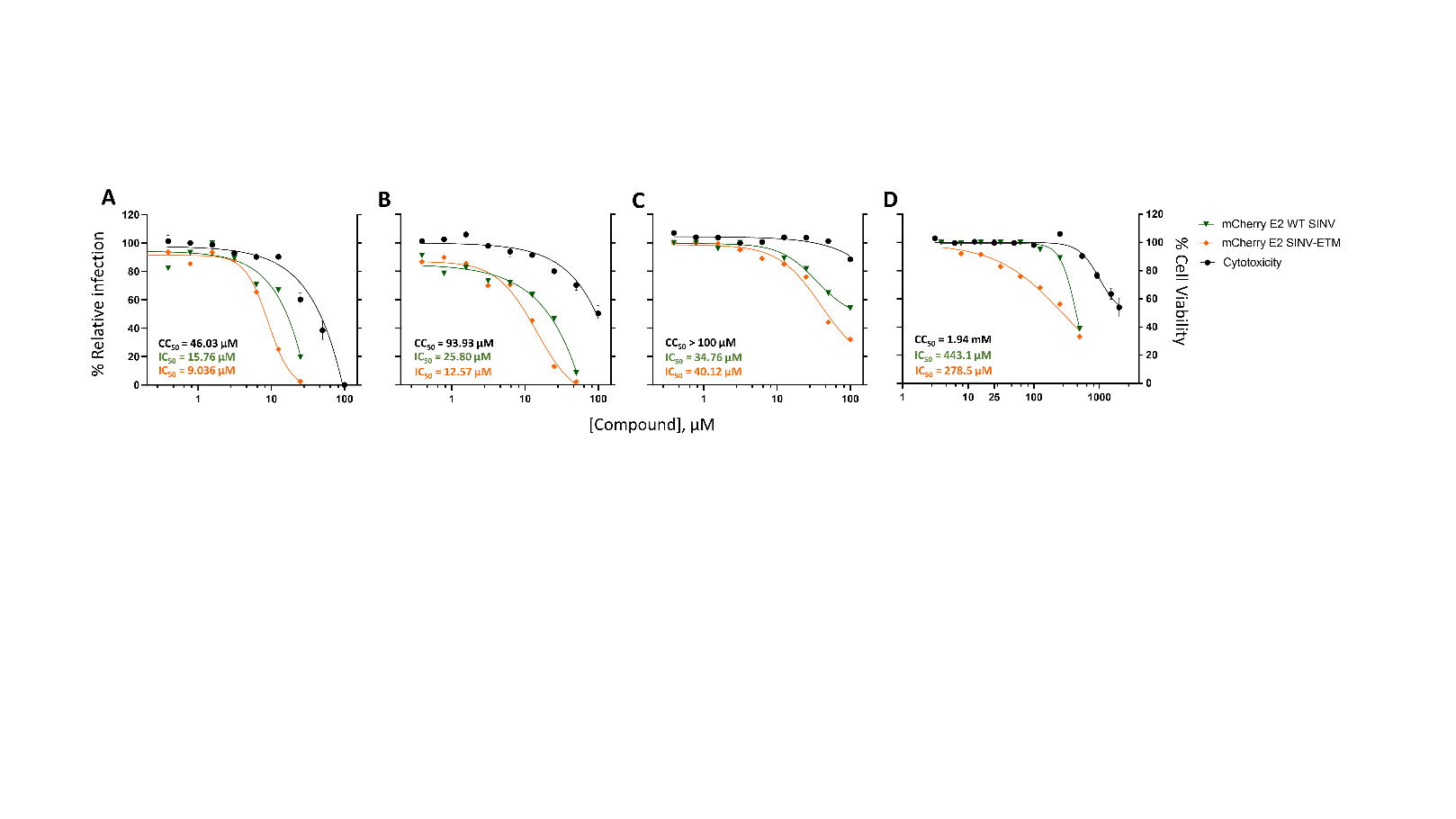


**Figure S4.** **Inhibition of SINV and SINV-ETM infection by amilorides and amantadine in BHK cells at MOI=0.1 and 24h incubation.** BHK cells were treated with indicated concentrations of (A) HMA, (B) EIPA, (C) DMA and (D) amantadine and infected with mCherry E2 WT SINV (green) and mCherry E2 SINV-ETM (orange) viruses at MOI=0.1 for 24 hours.


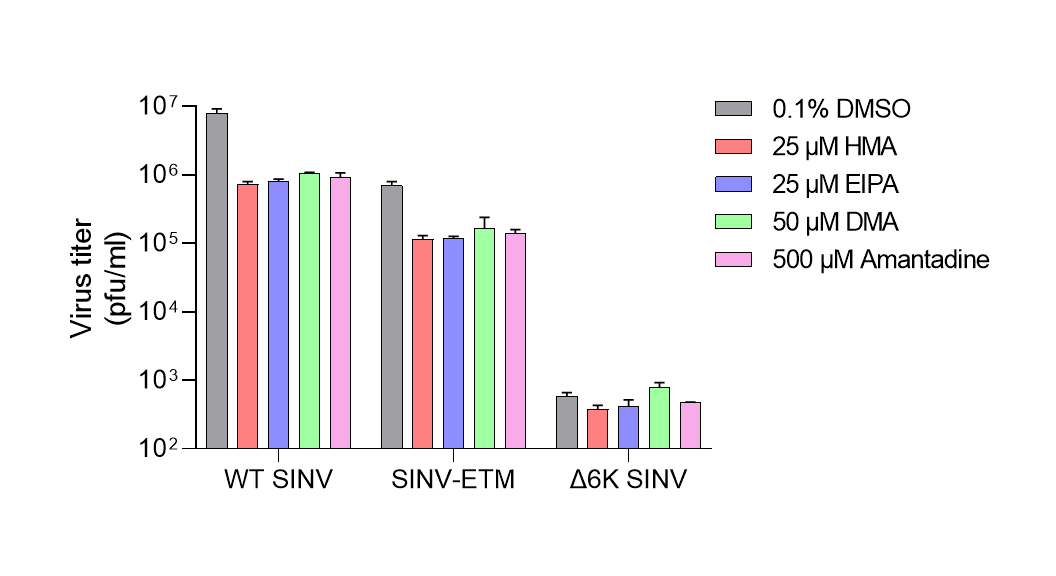


**Figure S5.** **Egress Inhibition assay.** BHK cells were infected with WT SINV, SINV-ETM, and ∆6K SINV at an MOI of 5.0 and treated with indicated concentrations of HMA, EIPA, DMA and amantadine after 8 hours of infection. Viral titers were determined using cell supernatants harvested at 12 hours post infection. The graph depicts data from two independent experiments.


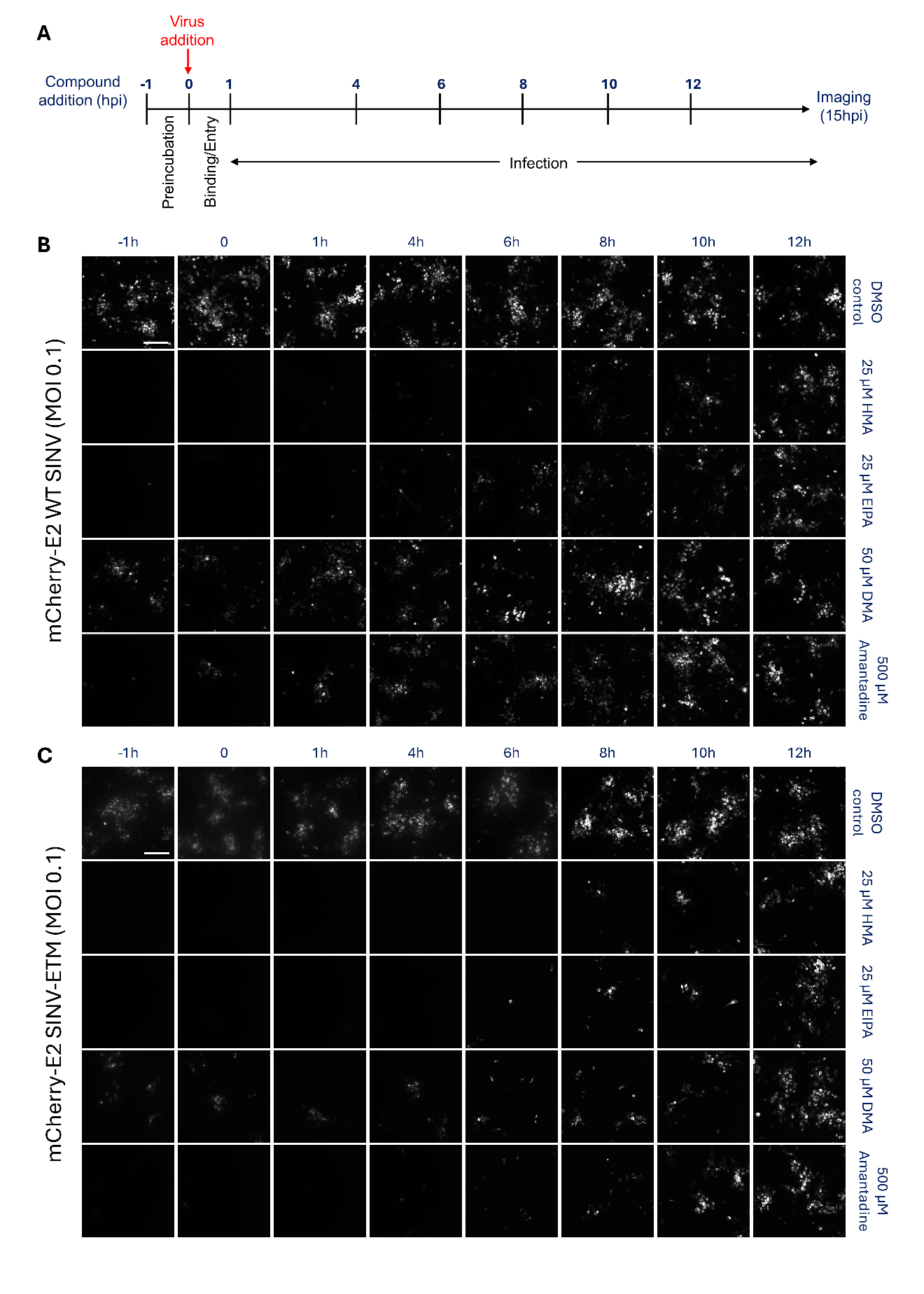


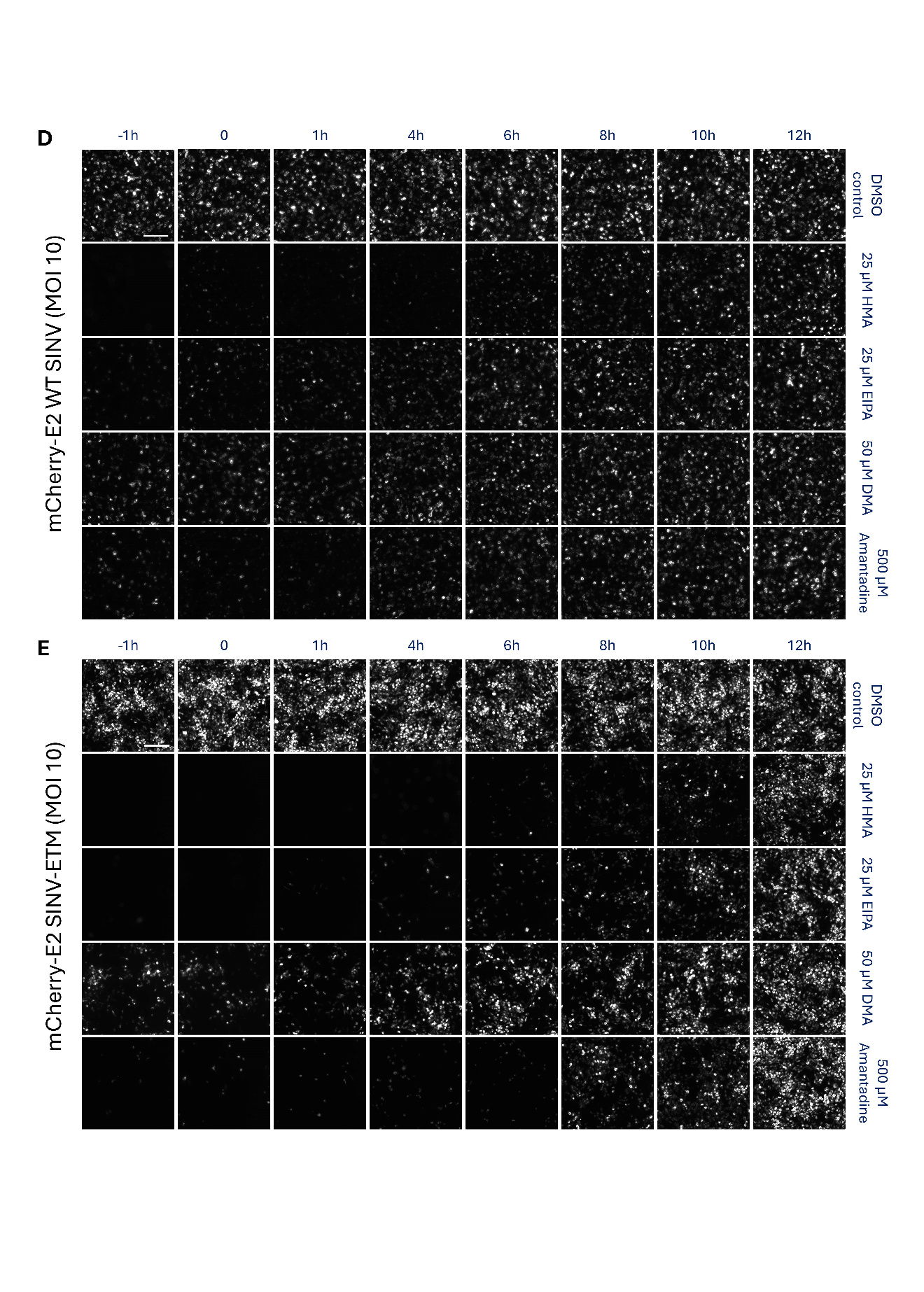


**Figure S6. Time of Addition assay.** (A) Schematic representation of time of addition assay protocol. (B-E) Representative grayscale images from two independent experiments of BHK-21 cells infected with mCherry-tagged virus and treated with compounds at the indicated time points. Cells were infected with SINV or SINV-ETM at MOI of 0.1 (B, C) and 10 (D, E) and fixed at 15 hours post infection. Images for mCherry signal (TRITC channel) at 20X magnification were taken. Scale bar = 200 μm.


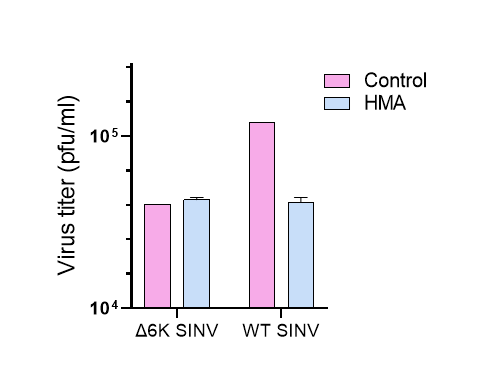


**Figure S7.** **Plaque Number Reduction assay.** WT SINV and ∆6K SINV, preincubated with HMA for 1 hour at 37°C, were filtered and plaqued on BHK cells. Pretreatment with HMA resulted in 0.2-0.3 log reduction in the titer of WT SINV relative to PBS-treated control but no change was seen in the titer of ∆6K SINV. The data is representative of two independent experiments.
